# DNA-based diagnosis of rare diseases in veterinary medicine: a 4.4 kb deletion of ITGB4 is associated with epidermolysis bullosa in Charolais cattle

**DOI:** 10.1186/s12917-015-0366-0

**Published:** 2015-03-04

**Authors:** Martin Peters, Irene Reber, Vidhya Jagannathan, Barbara Raddatz, Peter Wohlsein, Cord Drögemüller

**Affiliations:** Chemisches und Veterinäruntersuchungsamt Westfalen, Zur Taubeneiche 10-12, 59821 Arnsberg, Germany; Institute of Genetics, Vetsuisse Faculty, University of Bern, Bremgartenstrasse 109a, 3001 Bern, Switzerland; Department of Pathology, University of Veterinary Medicine Hannover, Bünteweg 17, 30559 Hannover, Germany

**Keywords:** Cattle, Rare genetic disease, Skin fragility, Junctional epidermolysis bullosa, Whole genome sequencing, Integrin beta 4, ITGB4

## Abstract

**Background:**

Rare diseases in livestock animals are traditionally poorly diagnosed. Other than clinical description and pathological examination, the underlying causes have, for the most part, remained unknown. A single case of congenital skin fragility in cattle was observed, necropsy, histological and ultrastructural examinations were carried out and whole genome sequencing was utilized to identify the causative mutation.

**Results:**

A single purebred female Charolais calf with severe skin lesions was delivered full-term and died spontaneously after birth. The clinical and pathological findings exactly matched the gross description given by previous reports on epitheliogenesis imperfecta and epidermolysis bullosa (EB) in cattle. Histological and ultrastructural changes were consistent with EB junctionalis (EBJ). Genetic analysis revealed a previously unpublished ITGB4 loss-of-function mutation; the affected calf was homozygous for a 4.4 kb deletion involving exons 17 to 22, and the dam carried a single copy of the deletion indicating recessive inheritance. The homozygous mutant genotype did not occur in healthy controls of various breeds but some heterozygous carriers were found among Charolais cattle belonging to the affected herd. The mutant allele was absent in a representative sample of unrelated sires of the German Charolais population.

**Conclusion:**

This is the first time in which a recessively inherited ITGB4 associated EBJ has been reported in cattle. The identification of heterozygous carriers is of importance in avoiding the transmission of this defect in future. Current DNA sequencing methods offer a powerful tool for understanding the genetic background of rare diseases in domestic animals having a reference genome sequence available.

**Electronic supplementary material:**

The online version of this article (doi:10.1186/s12917-015-0366-0) contains supplementary material, which is available to authorized users.

## Background

Rare or so-called orphan diseases, which affect only a very small number of individuals, have been identified in both humans and domestic animal species. The majority of rare diseases are caused by altered functions of single genes. Although the individual diseases are rare, collectively they are common, affecting millions of people worldwide [1]. In non-laboratory animals, the number of rare genetic diseases is unknown, but Online Mendelian Inheritance in Animals (OMIA), a catalogue of inherited disorders and associated genes in animals, reports more than 2500 phenotypes in eleven domestic animal species [2]. Currently, the gene mutation responsible for approximately 20% of rare diseases in domestic animals has been determined [2]. This has been accomplished over the past 25 years either by the targeted analysis of individual candidate genes or labor- and resource-intensive positional cloning approaches, such as linkage mapping or genome-wide association studies [3]. For this purpose, a series of cases showing an identical phenotype was needed [4]. The advent of next-generation sequencing technology, in combination with the establishment of a reference genome sequence for domestic animal species, such as for the bovine genome in 2009 [5], have changed the prospects enormously [6]. Today, studying the molecular aetiology of single cases is also feasible, e.g., in cattle [6,7], as has been successfully carried out in humans for approximately five years now [1].

Congenital skin fragility, also called epidermolysis bullosa (EB), represents a heterogeneous group of rare diseases reported in different species, including livestock animals. In the majority of cases it is genetically determined and, in humans, 18 EB-associated genes have currently been identified which encode the structural proteins involved in epidermal and dermal adhesion [8]. Various EB forms have been described in cattle [9-16], but the associated genes (KRT5 and COL7A1) have been identified for only two outbreaks of recessively inherited EB forms (OMIA 000340–9913 and OMIA 000341–9913) [13,16]. As is known for EB in other domestic animals, these two bovine EB diseases were genetically characterised by analysing well-known EB candidate genes [16,17].

A single case of severe congenital EB was observed in Charolais cattle. The purpose of this study was to characterise the phenotype in comparison to the known EB forms of different species. In parallel, a whole genome sequencing-based mutation analysis was carried out focusing on known EB candidates, and an associated loss-of-function mutation in the integrin beta 4 (ITGB4) gene was detected.

## Results

### Phenotype description

**Figure 1:**
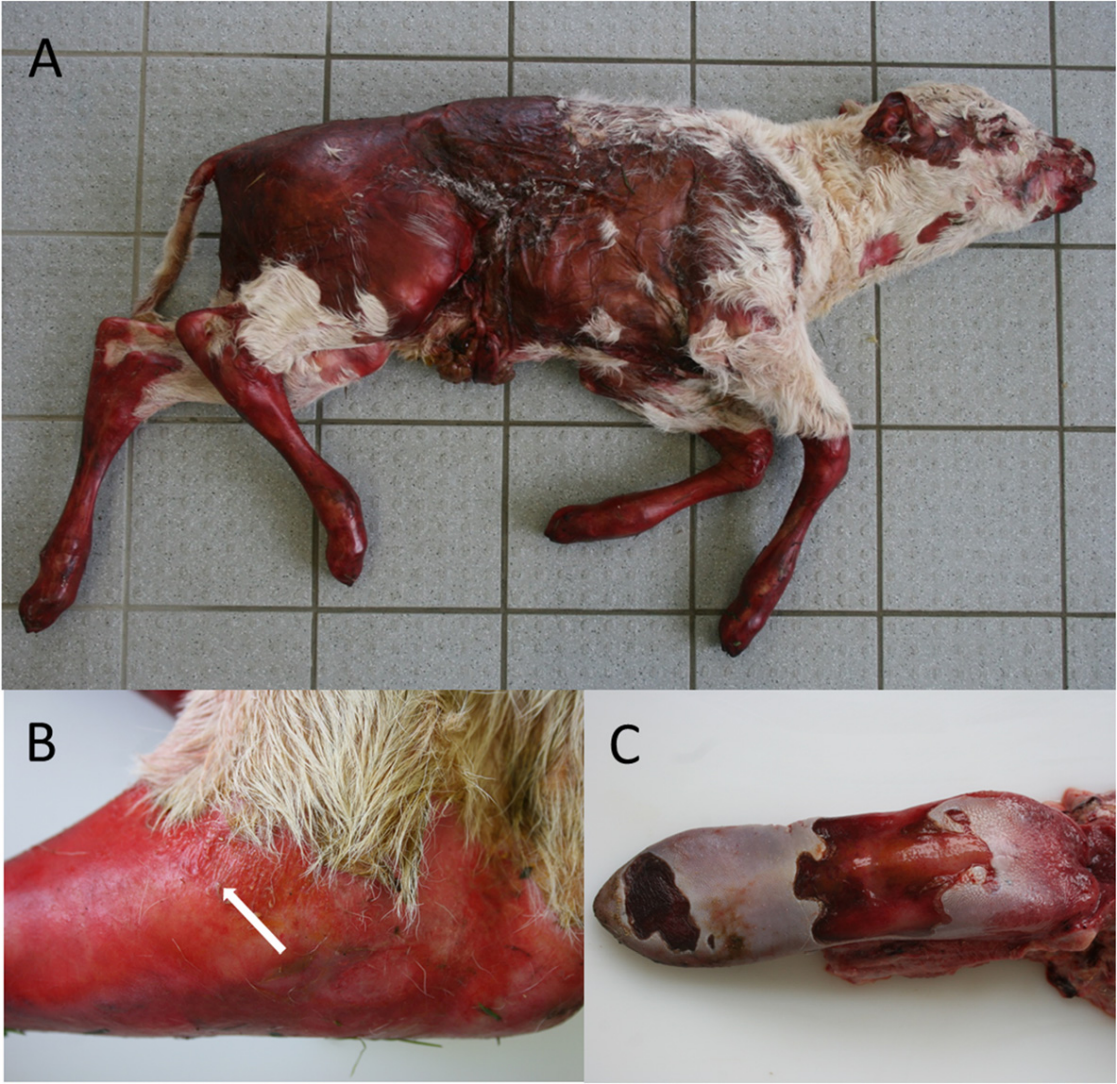
**Epidermolysis bullosa in a female Charolais calf. (A)** Note the extensive epidermal loss at the trunk, ears, distal limbs and muzzle as well as exungulation of the claws. **(B)** Multiple cutaneous vesicles (arrow) at the transition area between alopecic and haired skin. **(C**) Extensive mucosal defects of the tongue.

A single purebred, female Charolais calf of 22.4 kg was delivered full-term and died immediately after birth. The calf underwent post-mortem examination at the Chemisches und Veterinäruntersuchungsamt Westfalen (Northrhine Westphalia, Germany). The calf exhibited multifocally extensive alopecia, erosions and ulcers on the rump, head, and external surfaces of the pinnae, eyelids, nose, muzzle, lips and distal extremities with onychomadesis of all four feet (Figure 1A). Parts of the small intestine protruded through the navel. The hairless skin showed crusts and multiple small vesicles at the border with the haired skin (Figure 1B). There were severe oral lesions with focally missing cutaneous mucous membranes of the gingiva, hard palate, and on the back (Figure 1C) and ventral aspect of the tongue. There were linear skin defects at the anal- and vulvocutaneous junctions. No other gross lesions were detected.

### Histopathological and ultrastructural findings

**Figure 2:**
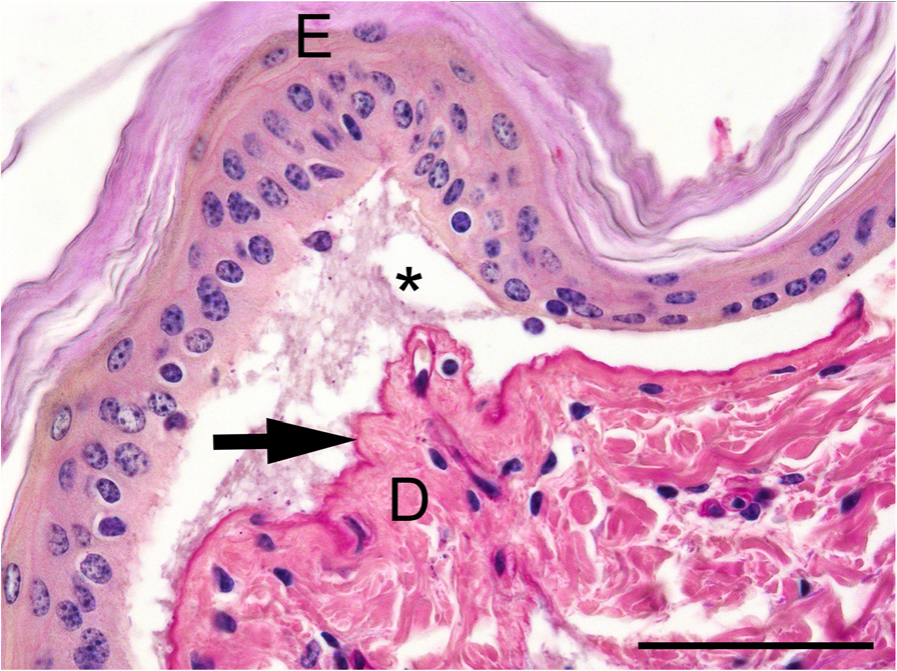
**Micrograph of the affected skin.** Macroscopically unaffected skin from the left hind leg of a Charolais calf having a subepidermal cleft formation with acellular, proteinaceous fluid (asterisk): the PAS-positive basement membrane (arrow) is located at the floor of the cleft attached to the adjacent dermis. E = epidermis; D = dermis. Periodic acid-Schiff (PAS)-reaction. Bar = 25 μm.

**Figure 3:**
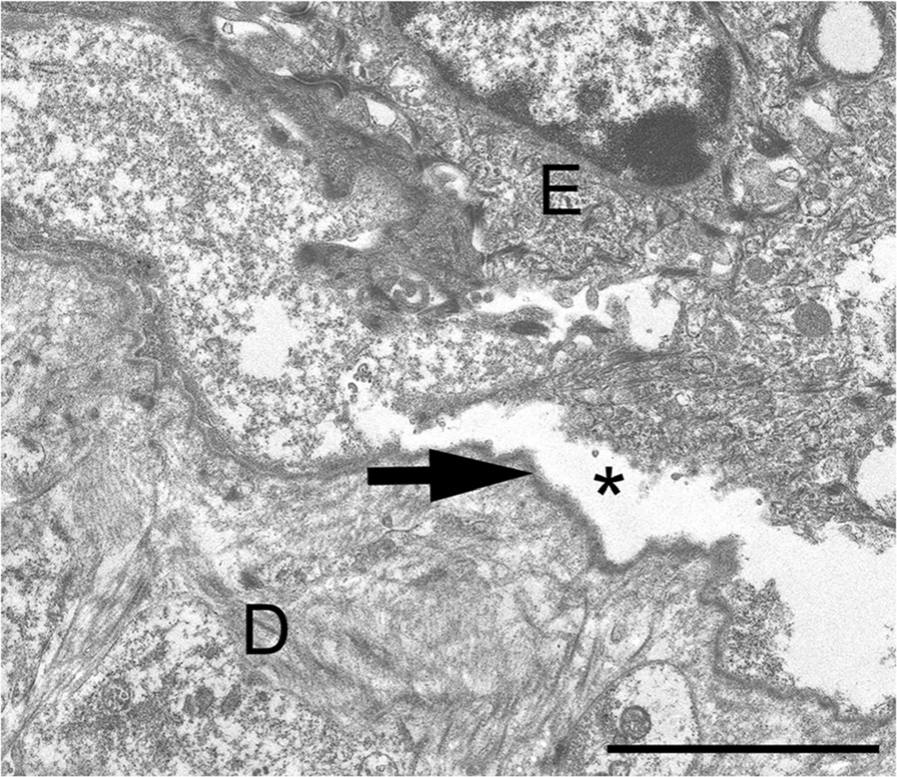
**Transmission electron microscopy of the skin.** Macroscopically unaffected skin from the left hind leg with a severe subepidermal cleft formation (asterisk) located in the lamina lucida of the basement membrane. The lamina densa (arrow) is attached to the dermis. E = epidermis; D = dermis. Bar = 250 nm.

Histological examination of the affected skin lesions or oral mucous membranes multifocally confirmed a complete loss of the epidermis or the epithelium which was covered by serocellular crusts. The underlying dermis or submucosa multifocally showed mild to moderate, acute, diffuse haemorrhages and a mild infiltration of neutrophils and mononuclear cells. The adjacent skin or mucous membranes displayed severe, subepidermal or subepithelial cleft formations of various lengths occasionally filled with eosinophilic, proteinacious fluid, cellular debris, haemorrhage and single neutrophils. The cleft formation extended around the hair follicles in varying degrees (Figure 2). periodic acid-Schiff (PAS)-reaction identified the basement membrane associated with the floor of the cleft. Consequently, the cleft formation was located between the basal layers of the epithelial cells and the basement membrane. Ultrastructurally, the cleft formation was located in the lamina lucida of the basement membrane. The lamina densa was attached to the dermis (Figure 3).

### Mutation analysis

The dam of the affected calf showed no clinically visible skin anomalies. In addition, the owner did not observe any similar congenitally malformed newborns in his herd. Unfortunately, the identity of the sire could not be determined as the farmer keeps more than 200 cows and some natural service sires which were used simultaneously had already been slaughtered. Since it was difficult to predict whether the disease was dominantly or recessively inherited without family information, we hypothesised two different possible scenarios: either a fully penetrant dominant acting *de novo* mutation which occurred in a single parental gamete or happened during early embryonic development of the calf, or a recessively inherited mutation present in the homozygous state transmitted by both parents due to inbreeding.

**Figure 4:**
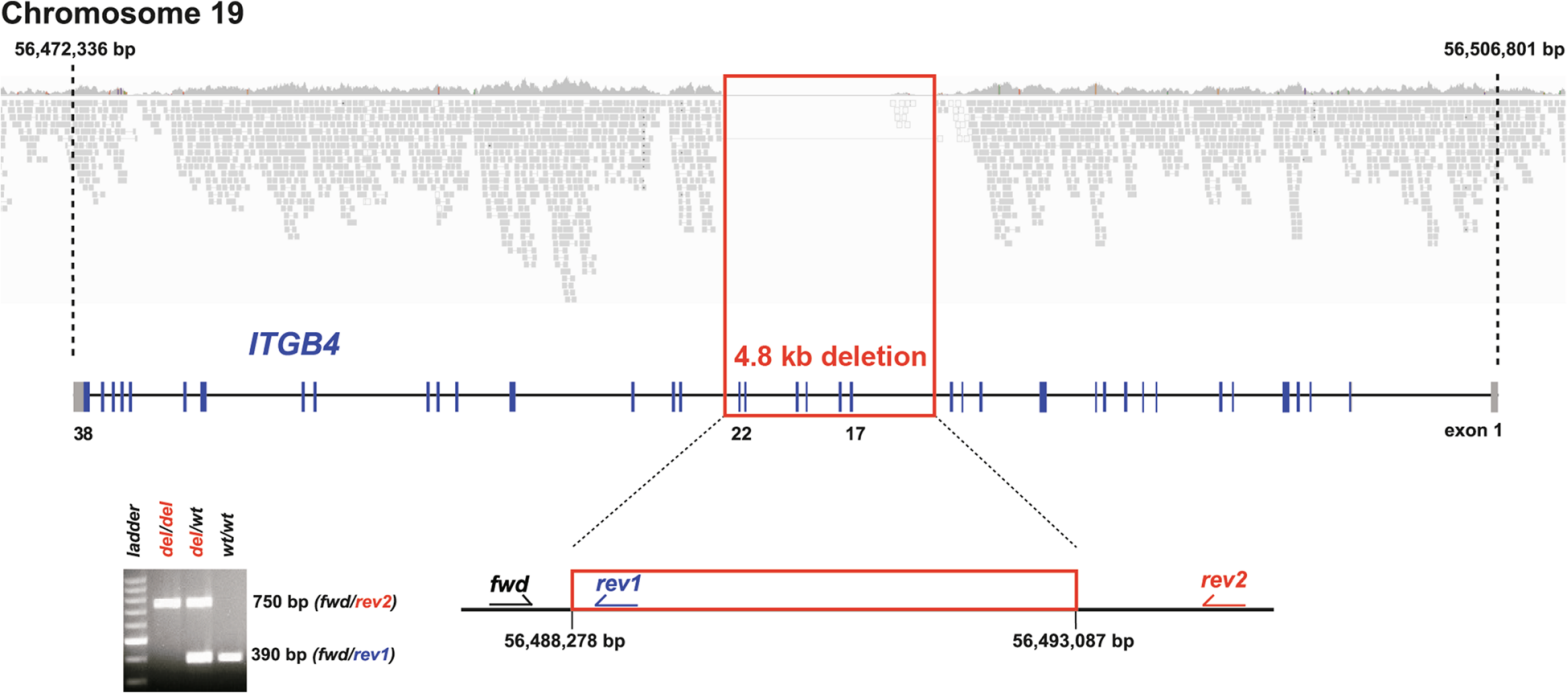
**Genetic characterisation of the ITGB4 mutation.** Whole genome sequencing of the affected calf (shown above) revealed the presence of a homozygous 4809 bp sized deletion on cattle chromosome 19 (shown in red). The deleted segment contains the coding exons 17 to 22 of the ITGB4 gene (shown in blue). Note that, taking into account the gap in the reference sequence, the actual size of the deletion is 4405 bp. A diagnostic PCR performed on genomic DNA using a combination of three allele-specific primers allows genotype differentiation (shown below). The gel picture shows the affected calf (*del*/*del*), its heterozygous dam (*del*/*wt*) and a normal control (*wt*/*wt*).

First, a recent dominant *de novo* mutation was hypothesised and the entire genome of the affected animal was therefore sequenced in order to detect all the variants in the known EB comparative candidate genes. A total of 203,557,590 100 bp paired-end reads were collected from a shotgun fragment library corresponding roughly to a 14.3 fold coverage of the genome. The single nucleotide variants, and short insertions or deletions were called and compared to the reference genome and 68,729 high quality variants across the entire exome, including untranslated regions and 10 bp of flanking introns, were detected. The variants were additionally compared with 50 cow genomes of various breeds which had been sequenced in our laboratory in the course of other ongoing studies. Assuming that the causative variant would be completely absent in these controls but present in the affected calf, a total of 981 variants, of which 955 were coding variants, occurred privately only in the EB-affected calf and were not present in any control. For these variants, a total of 1458 effects on annotated genes and loci were predicted (Additional file 1). Of the 955 coding variants, 745 were present in the heterozygous state and 322 in the homozygous state. This analysis revealed no exomic sequence variants located within one of the 18 EB candidate genes. In addition, larger deletions in the sequenced case and in 10 control cow genomes with a genome-wide coverage of more than 10 fold were searched for. A total of 890 deletions were private deletions occurring only in the genome of the affected Charolais animal in which a single 4.8 kb deletion was detected in the region of one of the comparative candidate genes (ITGB4), starting at position 56,488,275 on cattle chromosome 19 (UMD3.1/bosTau6 assembly). Visual inspection of the mapped sequence reads confirmed the presence of a large (4809 bp) deletion in the ITGB4 gene affecting six coding exons (c.1,765-1,863_2,613-2,636del) with breakpoints in intron 16 and intron 22 (Figure 4). The presence of the genomic deletion in the homozygous state was confirmed by polymerase chain reaction (PCR) and agarose gel electrophoresis (Figure 4). To prove and define the precise breakpoints of this deletion, the obtained PCR products were sequenced. In this way, it was possible to sequence a previously uncharacterised sequence gap in the bovine reference sequence which was present in intron 16, revealing that this region is 404 bp shorter than presented in the reference sequence (Additional file 2). This detailed analysis finally showed that the exact size of the deletion on chromosome 19 was 4405 bp (4809 bp minus 404 bp).

**Figure 5:**
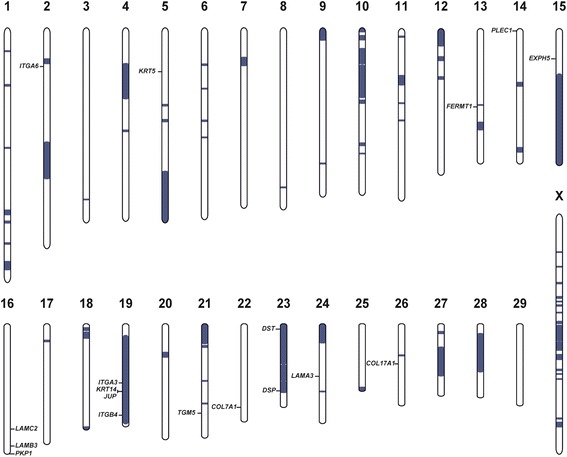
**Genome-wide homozygosity mapping across the genome of the affected cattle and the cattle genome position of 18 known genes associated with skin fragility disorders.** Note that 6 candidates genes are co-localised with the 80 largest (>1 Mb) homozygous blocks detected (shown in blue).

In addition, under the assumption of a possible recessive mutation, it was decided to apply a homozygosity mapping approach to determine the homozygous regions in the genome of the affected calf. It was hypothesised that the affected animal would be identical by descent for the causative mutation and flanking chromosomal segments due to parents which shared a common ancestor. The genotypes of 777,962 single nucleotide polymorphisms (SNPs) were analysed and the genotypes were checked for extended regions of homozygosity. A total of 80 genomic regions larger than 1 Mb were located on different cattle chromosomes (Figure 5). The largest homozygous region by far was located on cattle chromosome 19, containing 16,393 SNP markers and corresponding to a 54.7 Mb interval from 7.6 to 62.3 Mb (Figure 5). Within this genomic segment, 4 of the 18 known EB comparative candidate genes were located in the bovine genome including the ITGB4 gene which contains the above mentioned large genomic deletion. To experimentally prove the inheritance of this deletion, a diagnostic PCR was designed (Figure 4). This analysis confirmed the homozygous genotype of the affected calf and showed that the dam was carrying a single copy of the deletion (Figure 4). As expected, normal controls showed a single band of 390 bp, the homozygous affected calf showed a single band of 750 bp and the heterozygous dam showed both PCR products. Genotyping was carried out on a total of 162 Charolais cattle belonging to the herd into which the affected calf was born. This revealed that the homozygous mutant genotype was absent in all animals but we identified a total of 15 heterozygous carriers including the maternal grandmother of the affected calf. Due to missing detailed pedigree records we were not able to identify a possible common ancestor among the disease allele carriers. Finally, a total of 88 unrelated Charolais sires which were used for artificial insemination in Germany and 50 controls from various breeds were tested negatively for the presence of the ITGB4 deletion.

## Discussion

The skin fragility phenotype observed in a Charolais calf resembles previous reports of epidermolysis bullosa and epitheliogenesis imperfecta in calves [9-16]. Since epitheliogenesis imperfecta is not genetically defined and an in-depth pathological examination is necessary for distinction, both conditions might have been confused in the past and recorded in veterinary literature. Epidermolysis bullosa is a mechanobullous disorder and is classified into three groups according to the ultrastructural location of the blistering between the epidermis and the dermis. In EB simplex, the blister formation tissue separation occurs within the epidermal basal keratinocytes adjacent to the basal lamina. In junctional EB, the blister formation arises within the lamina lucida of the basal lamina. In dystrophic EB, the blister formation takes place at the dermal side of the basal lamina below the lamina densa. Which subtype of EB develops and how severe the lesions are depend on the functional defects of particular proteins. All three types of EB have been reported in cattle [17]. Ultrastructurally, the case reported in a Charolais calf could clearly be identified as EB junctionalis (EBJ). There is high morphologically agreement with another case of EBJ in this breed [18] in which, based on immuno-histological examination, a deficient expression of integrin α6β4 was hypothesised as being a possible cause. The identification of a loss-of-function mutation in the bovine ITGB4 gene could finally confirm this suspicion since the current case belongs to the same breed. In regard to the recessive inheritance of the deletion identified one can hypothesise that the mutation was possibly already present more than ten years ago in the French Charolais population. A likely scenario could be that, due to the importation of semen or living sires which were heterozygous for the mutation, the defective allele was introgressed into the German Charolais population. Alternatively, it could be that the formerly reported French case was caused by an independent ITGB4 mutation.

Mutations in ITGB4 are known to cause EBJ in humans [8]. The integrins are cell membrane receptors composed of alpha and beta subunits which orchestrate adhesive events in all tissues of the body. In the skin, they play an essential role in the architecture of the hemidesmosomes which mediate the stable attachment of the basal epithelial cells to the underlying basement membrane [19]. Due to the severity of EBJ in this case, a very likely causative mutation in the coding region of one of the well-characterised candidate genes was hypothesised. The approach of whole genome sequencing allowed the consideration of two possible scenarios: a recent dominant acting *de novo* mutation or a recessively inherited mutation which had already occurred some generations ago. That the EBJ phenotype could be explained by a recessive ITGB4 deletion which was very likely responsible for a similar case already one decade ago was able to be shown [18]. The transcript of the mutant allele lacked information regarding a significant part of the encoded protein since the deletion led to a frameshift and a premature stop codon. It was therefore assumed that the mutant transcripts probably underwent nonsense mediated decay so that, in the final analysis, the deletion represented a loss-of-function mutation with a non-existing integrin protein in the epidermis of the affected animal. In humans, a broad spectrum of clinical and morphological EBJ manifestations exists associated with ITGB4 mutations [8]. They range from non-lethal forms with very mild skin features to severe lethal phenotypes [20]. To date, at least 69 different mutations in human ITGB4 have been reported [21]. Pyloric atresia, which found regularly in humans suffering from the altered synthesis of integrin α6β4, was not detected in either of the affected Charolais calves [8,18].

## Conclusions

This study presents a recessively inherited ITGB4 associated EBJ form in cattle. Selection against this candidate causative mutation can now be used to eliminate this genetic disorder from Charolais cattle in production systems. The results obtained showed that current DNA sequencing methods offer a powerful tool for understanding the genetic background of rare diseases in domestic animals with a reference genome sequence available.

## Methods

### Ethics statement

The study was conducted according to national and international guidelines for animal welfare. Permission was obtained from the cattle owner agreed for the samples to be used in the study. The data were obtained during diagnostic procedures which would have been carried out regardless. This is a very special situation in veterinary medicine. Since the data were from client-owned cattle which underwent veterinary exams, according to the legal definitions in Germany, no “animal experiment” took place.

### Histopathological examination

Samples of various locations of the skin, oral mucosa, tongue, brain, lung, heart, muscle, liver, spleen, kidney and intestine were fixed in 10% neutral buffered formalin, embedded in paraffin wax, sectioned at 3 μm, and stained with haematoxylin/eosin according to routine methods. Selected sections were stained with PAS-reaction

### Ultrastructural examinantion

For transmission electron microscopy skin and oral mucosa tissue were fixed in 2.5% glutaraldehyde/cacodylate buffer for 24 h, post-fixed in 1% osmium tetroxide, dehydrated in a graded series of alcohol and embedded in epon (Serva, Heidelberg, Germany). Sixty nm thick ultra-thin sections were contrasted with 2% aqueous uranyl acetate and lead citrate and examined with a Zeiss EM 10C electron microscope (Zeiss, Oberkochen, Germany).

### Animals and genotyping

Blood samples were taken from the affected animal and from a total of 162 animals belonging to the herd into which the affected calf was born. Genomic DNA was isolated using the DNeasy Blood & Tissue Kit (Qiagen, Hilden, Germany) according to the manufacturer’s protocol. In addition, archived DNA samples of 81 Charolais bulls and 50 animals from different cattle breeds were used for genotyping the ITGB4 deletion.

The genotyping of the affected animal was carried out using the BovineHD BeadChip (illumina, San Diego, USA), including 777,961 evenly distributed SNPs and standard protocols as recommended by the manufacturer.

### Whole genome sequencing and variant calling

A fragment library with 300 bp insert size was prepared and one lane of illumina HiSeq2000 paired-end reads (2 × 100 bp) were collected. The reads were mapped to the cow reference genome UMD3.1/bosTau6 and aligned using Burrows-Wheeler Aligner (BWA) version 0.5.9-r16 [22] with default settings. The SAM file generated by BWA was then converted to BAM and the reads were sorted by chromosome using samtools [23]. The PCR duplicates were marked using Picard tools [24]. The Genome Analysis Tool Kit (GATK version 2.4.9) [25], was used to carry out local realignment and to produce a cleaned BAM file. Variant calls were then made with the unified genotyper module of GATK. The variant data for each sample were obtained in variant call format (version 4.0) as raw calls for all samples and sites flagged using the variant filtration module of GATK. Variant filtration was performed, following the best practice documentation of GATK version 4. The snpEFF software [26] together with the UMD3.1/bosTau Ensembl annotation was used to predict the functional effects of the variants detected. The pindel package using split-read approaches to identify large deletions and medium-size insertions in pair-end reads was used to detect structural variants in cleaned BAM files [27]. Hence, in order to avoid missing large inserts, deletions and false positives of all the variants detected in the region of EB genes (Additional file 3) were also manually inspected using the Integrative Genomics Viewer (IGV) [28].

The variants of a total of 50 genomes from various cattle breeds (14× Holstein, 6× Simmental, 5× Angler, 4× Brown Swiss, 3× Hinterwalder, 3× Vorderwalder, 2× Galloway, 2× Eringer, 2× Romagnola, 2× Scotish Highland Cattle, 2× Tyrolean Grey Cattle, 1× Hereford, 1× Limousin, 1× Pezzata Rossa Italiana, 2× crossbred), which had been sequenced in our laboratory in the course of other ongoing studies, were used as controls during filtering.

### Genetic testing

Primers for the amplification of the deletion were designed using the Primer3 software [29] after masking repetitive sequences with RepeatMasker [30]. The positions of the three primers used for genotyping (fwd GTGAGGGCTTCGTATGGGTA; rev 1 TGAACGAGGTGTACCGACAA; rev 2 AGTCGCTCTACACGGACACC) are displayed in Figure 4. Sanger sequencing was used to confirm the illumina sequencing results. For these experiments, PCR products using AmpliTaqGold360Mastermix (Life Technologies, Darmstadt, Germany) were amplified. The PCR products were loaded on 2% agarose gels for visual inspection of band size. The PCR products were directly sequenced on an ABI3730 capillary sequencer (Life Technologies) after treatment with exonuclease I and shrimp alkaline phosphatase. The sequence data were analysed using Sequencher 5.1 software (GeneCodes, Ann Arbor, USA).

### Availability and requirements

The genome data were made available freely at the European Nucleotide Archive [ENA:PRJEB7528] [31]. Further supporting data are included as additional files.

## Additional files

Additional file 1:
**Private exome variants of the affected calf.** A list of 981 DNA variants with 1471 predicted effects on annotated genes and loci. Additional file 2:
**Sequence details of the disease-associated deletion in the bovine ITGB4 gene.** The genomic sequence of the wild-type sequence surrounding the detected mutation on chromosome 19 is displayed. The upper line corresponds to the reference sequence of the UMD3.1 assembly and the lower line to the experimentally verified, shorter sequence. The 4405 bp deletion is indicated by a frame showing the precise breakpoints. Additional file 3:
**Candidate genes for epidermolysis bullosa (EB).** A list of 18 genes and their annotated position in the bovine genome.

## Abbreviations

COL7A1: Collagen 7A1
EB: Epidermolysis bullosa
EBJ: Epidermolysis bullosa junctionalis
ITGB4: Integrin beta 4
KRT5: Keratin 5
OMIA: Online Mendelian Inheritance in Animals
SNP: Single nucleotide polymorphism
