# Supplementary material for: DNA-based diagnosis of rare diseases in veterinary medicine: a 4.4 kb deletion of ITGB4 is associated with epidermolysis bullosa in Charolais cattle
Source: BMC Vet Res. 2015 Mar 4;11:48. doi: 10.1186/s12917-015-0366-0 (PMC4351973; doi:10.1186/s12917-015-0366-0)
Supplement: Additional file 2: — Sequence details of the disease-associated deletion in the bovine ITGB4 gene. The genomic sequence of the wild-type sequence surrounding the detected mutation on chromosome 19 is displayed. The upper line corresponds to the reference sequence of the UMD3.1 assembly and the lower line to the experimentally verified, shorter sequence. The 4405 bp deletion is indicated by a frame showing the precise breakpoints. [file 12917_2015_366_MOESM2_ESM.pdf]

**UMD3.1/bosTau6 assembly**
**Chr.19: Pos. 56488001 to 56488277**

```
AGAAGTGTGTCACCCACTGGACCATCTGGTTGGTAACCAGCGGCGCCTCCAGGATCAAGG
||||||||||||||||||||||||||||||||||||||||||||||||||||||||||||
AGAAGTGTGTCACCCACTGGACCATCTGGTTGGTAACCAGCGGCGCCTCCAGGATCAAGG

CCTTTGGCCTCAGACGAGGGCTGGCTCTGGGCACCGGCTATCCCTGGTACTGGGTGGGAG
||||||||||||||||||||||||||||||||||||||||||||||||||||||||||||
CCTTTGGCCTCAGACGAGGGCTGGCTCTGGGCACCGGCTATCCCTGGTACTGGGTGGGAG

CTATTGCCCGTGTGAGGCCAGGGACTGGGTTGGCTCGTTTCCCTGTGGGTTTCTTATTCT
||||||||||||||||||||||||||||||||||||||||||||||||||||||||||||
CTATTGCCCGTGTGAGGCCAGGGACTGGGTTGGCTCGTTTCCCTGTGGGTTTCTTATTCT

CATACAGGGGCTGGCTCTCGGTGAGGGCTTCGTATGGGTATGTGTTCAGTGAAGGTCCTA
||||||||||||||||||||||||||||||||||||||||||||||||||||||||||||
CATACAGGGGCTGGCTCTCGGTGAGGGCTTCGTATGGGTATGTGTTCAGTGAAGGTCCTA

CCCCAGGACAGACCCAGACAGCCCTCCTCTCCCATGG
|||||||||||||||||||||||||||||||||||||
CCCCAGGACAGACCCAGACAGCCCTCCTCTCCCATGG
```

**Chr.19: Pos.56488278 to 56793087 = 4809bp - 404bp = 4.4kb deletion**

```
GGCGGAGATGATGGAGATGGAAGTGGGGATGCGGGAGGTGGGGATAGGGGAGGTGGGACT
||||||||||||||||||||||||||||||||||||||||||||||||||||||||||||
GGCGGAGATGATGGAGATGGAAGTGGGGATGCGGGAGGTGGGGATAGGGGAGGTGGGACT

TGGGGCTGGTACTGCCCACGGATGTGTCTTCGACTCACTTTTTCCCGGCGTTGGGCTGCT
||||||||||||||||||||||||||||||||||||||||||||||||||||||||||||
TGGGGCTGGTACTGCCCACGGATGTGTCTTCGACTCACTTTTTCCCGGCGTTGGGCTGCT

GCCTGTGGGGGACAAAGTGAAGACTGTGAGCCAGATGGTCCCTAGGGGGCAAGGATTATC
||||||||||||||||||||||||||||||||||||||||||||||||||||||||||||
GCCTGTGGGGGACAAAGTGAAGACTGTGAGCCAGATGGTCCCTAGGGGGCAAGGATTATC

TGCTTGGGAGAAATCCCCTGGCATCAGCTATGGGAGCAGCGCCCCCCCCAACCAACCCCA
||||||||||||||||||||||||||||||||||||||||||||||||||||||||||||
TGCTTGGGAGAAATCCCCTGGCATCAGCTATGGGAGCAGCGCCCCCCCCAACCAACCCCA

CCCCGGGCGCCTAGGACCCACCGGAACTTGGTCTGCTGGAGGTTGTGAGCGCCTGTGATT
||||||||||||||||||||||||||||||||||||||||||||||||||||||||||||
CCCCGGGCGCCTAGGACCCACCGGAACTTGGTCTGCTGGAGGTTGTGAGCGCCTGTGATT

TGTCGGTACACCTCGTTCAGCTGCCAGGCAAGGGAGGGCTGTGAGGCTGGGGCCCTGGGG
||||||||||||||||||||||||||||||||||||||||||||||||||||||||||||
TGTCGGTACACCTCGTTCAGCTGCCAGGCAAGGGAGGGCTGTGAGGCTGGGGCCCTGGGG

GAGCCCCTGCACCCAGAGGACCTCCTGGGCATGGCTGGGGGTGTGGCCACAGGCATGTGC
||||||||||||||||||||||||||||||||||||||||||||||||||||||||||||
GAGCCCCTGCACCCAGAGGACCTCCTGGGCATGGCTGGGGGTGTGGCCACAGGCATGTGC

AGTGGCTCTACCCTTCTGGAAGCATTCATGTGGGGCAGATCCCAGGGGACCCCCCGGCCA
||||||||||||||||||||||||||||||||||||||||||||||||||||||||||||
AGTGGCTCTACCCTTCTGGAAGCATTCATGTGGGGCAGATCCCAGGGGACCCCCCGGCCA

CACGTGTGTCCGAGAAAGCCCTTCTTTCCCACCCAAGGCGGCTTCCCTCTGNNNNNNNNN
|||||||||||||||||||||||||||||||||||||||||||||||||||:::::::::
CACGTGTGTCCGAGAAAGCCCTTCTTTCCCACCCAAGGCGGCTTCCCTCTGCCCTCCTGC
```

```
NNNNNNNNNNNNNNNNNNNNNNNNNNNNNNNNNNNNNNNNNNNNNNNNNNNNNNNNNNNN
::::::::::::::::::::::::::::::::::::::::::::::::::::::::::::
CACCCAGCTCGCACCCGGCCCAGAGGTGCCCACTGCCTGGGACCGGCTCCTTACATTCTC

NNNNNNNNNNNNNNNNNNNNNNNNNNNNNNNNNNNNNNNNNNNNNNNNNNNNNNNNNNNN
::::::::::::::::::::::::::::::::::::::::::::::::::::::::::::
CTCCACCTCCTGGCGCAGCTGGTCGCATTCTCGCGTGCCAGGCTTCAGCAGGTTCTCGGT

NNNNNNNNNNNNNNNNNNNNNNNNNNNNNNNNNNNNNNNNNNNNNNNNNNNNNNNNNNNN
:::::::::::::::::::::::::::::::::::::
GCAGAGGCGAGCAAGCCGCAGGGACAGTCCGTAGGGC-----------------------

NNNNNNNNNNNNNNNNNNNNNNNNNNNNNNNNNNNNNNNNNNNNNNNNNNNNNNNNNNNN

------------------------------------------------------------

NNNNNNNNNNNNNNNNNNNNNNNNNNNNNNNNNNNNNNNNNNNNNNNNNNNNNNNNNNNN

------------------------------------------------------------

NNNNNNNNNNNNNNNNNNNNNNNNNNNNNNNNNNNNNNNNNNNNNNNNNNNNNNNNNNNN

------------------------------------------------------------

NNNNNNNNNNNNNNNNNNNNNNNNNNNNNNNNNNNNNNNNNNNNNNNNNNNNNNNNNNNN

------------------------------------------------------------

NNNNNNNNNNNNNNNNNNNNNNNNNNNNNNNNNNNNNNNNNNNNNNNNNNNNNNNNNNNN

------------------------------------------------------------


NNNNNNNNNNNNNNNNNNNNNNNNNNNNNNNNNNNNNNNNNNNNNNNNNNNNNNNNNNNN

------------------------------------------------------------

NNNNNNNNNNNNNNNNNNNACTAGGGCAGGTGAGGAGGGGGGTCGTCAGTCCCGCCATGA
-------------------|||||||||||||||||||||||||||||||||||||||||
                   ACTAGGGCAGGTGAGGAGGGGGGTCGTCAGTCCCGCCATGA

TGGGCACCACAGAAGCCTCCTCCAGAAGCCGGGCTCGGGATTGGGGGGCACCGGGGGCGT
|||||||||||||||||||||||||||||||||||| |||||||||||||||||||||||
TGGGCACCACAGAAGCCTCCTCCAGAAGCCGGGCTC-GGATTGGGGGGCACCGGGGGCGT

GTACCCCTCAGGAAGGGTAGGAGGTGGCCAGCCCTGTGCCTGCACCCGATACCCAGAGAA
||||||||||||||||||||||||||||||||||||||||||||||||||||||||||||
GTACCCCTCAGGAAGGGTAGGAGGTGGCCAGCCCTGTGCCTGCACCCGATACCCAGAGAA

AAGAGGAATGCTGGATCCCAGACCGGGGAGCAGTGTGAATGTGCCCCGGGCCCCAAGGGC
||||||||||||||||||||||||||||||||||||||||||||||||||||||||||||
AAGAGGAATGCTGGATCCCAGACCGGGGAGCAGTGTGAATGTGCCCCGGGCCCCAAGGGC

AGCGCCAGGAGACGTGGTGCCCAGCCCCGGCCCTCACCCAGCTCTGAGGGGTTGATGCCT
||||||||||||||||||||||||||||||||||||||||||||||||||||||||||||
AGCGCCAGGAGACGTGGTGCCCAGCCCCGGCCCTCACCCAGCTCTGAGGGGTTGATGCCT

GCAGCGTGCGAGGCGAAGCCCGGCCGCTGCACGTTGTTGGTGATCTTCCATCGGACTGTG
||||||||||||||||||||||||||||||||||||||||||||||||||||||||||||
GCAGCGTGCGAGGCGAAGCCCGGCCGCTGCACGTTGTTGGTGATCTTCCATCGGACTGTG
```

```
TCGCGACCCTTGAGGTTCCCGCTGCGCAGCAAGGGCGTGTCCAAGTGGTCCGAGGCCATC
||||||||||||||||||||||||||||||||||||||||||||||||||||||||||||
TCGCGACCCTTGAGGTTCCCGCTGCGCAGCAAGGGCGTGTCCAAGTGGTCCGAGGCCATC

AGGTTCTCTCGCAGCATGTAATGGTCTTCCTTGAAGCCCACCATGTGACCTGTGGGACCT
||||||||||||||||||||||||||||||||||||||||||||||||||||||||||||
AGGTTCTCTCGCAGCATGTAATGGTCTTCCTTGAAGCCCACCATGTGACCTGTGGGACCT

GGGCCTCAGTACGGGGCTGAGGGGCTGCTTCCTAGACCCTCCCCCACCACACCCCACAGG
||||||||||||||||||||||||||||||||||||||||||||||||||||||||||||
GGGCCTCAGTACGGGGCTGAGGGGCTGCTTCCTAGACCCTCCCCCACCACACCCCACAGG

TGGGACCTCCACCGTCCCCAGCCTCTGTCCCAGACTTGGCACCCTGCTTCCCCTCCACCC
||||||||||||||||||||||||||||||||||||||||||||||||||||||||||||
TGGGACCTCCACCGTCCCCAGCCTCTGTCCCAGACTTGGCACCCTGCTTCCCCTCCACCC

CCGCTGAAGGAAGGAGCTGTCAGAAGTCCACCGCCCCCGCCGAGGCCAGGCCCGTACCTC
||||||||||||||||||||||||||||||||||||||||||||||||||||||||||||
CCGCTGAAGGAAGGAGCTGTCAGAAGTCCACCGCCCCCGCCGAGGCCAGGCCCGTACCTC

GGTTGCAGCAAGGGAGAAGGGCCAGGCAGGCCTAGGAGGCAAGGAGGGCGGGGATGAGAG
||||||||||||||||||||||||||||||||||||||||||||||||||||||||||||
GGTTGCAGCAAGGGAGAAGGGCCAGGCAGGCCTAGGAGGCAAGGAGGGCGGGGATGAGAG

CTGAGACGGCCGCACCGGCCCAGGGAAGTCCCTCCGGCCACCGCCTCCCACCCACGCCAG
||||||||||||||||||||||||||||||||||||||||||||||||||||||||||||
CTGAGACGGCCGCACCGGCCCAGGGAAGTCCCTCCGGCCACCGCCTCCCACCCACGCCAG

TGGTCCAGAGGGGTCAGCAGTGCCTTCCCCAGACACTGGGATATCCAGGCAGTTACAGGG
||||||||||||||||||||||||||||||||||||||||||||||||||||||||||||
TGGTCCAGAGGGGTCAGCAGTGCCTTCCCCAGACACTGGGATATCCAGGCAGTTACAGGG

GCCTGGGTGTGGGCACAGAGGCGAACAGGGGCAGAGGGTACCCAAAATGCATGCCATGGG
||||||||||||||||||||||||||||||||||||||||||||||||||||||||||||
GCCTGGGTGTGGGCACAGAGGCGAACAGGGGCAGAGGGTACCCAAAATGCATGCCATGGG

ACTCTGGTCCCGCTATCTACTCTGGAAAGGAAAAGGTTCTGTGATCAAACCCCTTTGGGA
||||||||||||||||||||||||||||||||||||||||||||||||||||||||||||
ACTCTGGTCCCGCTATCTACTCTGGAAAGGAAAAGGTTCTGTGATCAAACCCCTTTGGGA

AGCACTGAATTCCTTCCCCATGTAGTTTCCTTATGCAAACAGCTAGCTCAAAGGCTCCGA
||||||||||||||||||||||||||||||||||||||||||||||||||||||||||||
AGCACTGAATTCCTTCCCCATGTAGTTTCCTTATGCAAACAGCTAGCTCAAAGGCTCCGA

GAAGCCCTGCAGAGGAGAAACCGCTTTCTCAAAAGTGTTTGATCACAGAACGCTTTCTCC
||||||||||||||||||||||||||||||||||||||||||||||||||||||||||||
GAAGCCCTGCAGAGGAGAAACCGCTTTCTCAAAAGTGTTTGATCACAGAACGCTTTCTCC

CTGTAACACGCATGAATACGACCCTGGGGGCTGCCGTAGGGGCACCTCTGCCCCCTTCAG
||||||||||||||||||||||||||||||||||||||||||||||||||||||||||||
CTGTAACACGCATGAATACGACCCTGGGGGCTGCCGTAGGGGCACCTCTGCCCCCTTCAG

TATCCCACGCCAGGCCATGCTCTGCACTCTCCTGTCTCCGGTCTTTCTTCCCAGGGAGAT
||||||||||||||||||||||||||||||||||||||||||||||||||||||||||||
TATCCCACGCCAGGCCATGCTCTGCACTCTCCTGTCTCCGGTCTTTCTTCCCAGGGAGAT

GGCCAGCTCCTGGTGGGCAGAGCCTTTCCTTCTTGTTGGTTCTCGTGACCCTTTGCATGC
||||||||||||||||||||||||||||||||||||||||||||||||||||||||||||
GGCCAGCTCCTGGTGGGCAGAGCCTTTCCTTCTTGTTGGTTCTCGTGACCCTTTGCATGC
```

```
GGCTGACCCCCGGGCCAGGGACTCCCAGGAATCCAGCCACTGGTGGCTGGCAGAGGCGAG
||||||||||||||||||||||||||||||||||||||||||||||||||||||||||||
GGCTGACCCCCGGGCCAGGGACTCCCAGGAATCCAGCCACTGGTGGCTGGCAGAGGCGAG

GGGCTGGGGTGGGGACGGCACCAGAGCTGGGCGGAACGTGTGGGTTCCCTTGGCCTCTGC
||||||||||||||||||||||||||||||||||||||||||||||||||||||||||||
GGGCTGGGGTGGGGACGGCACCAGAGCTGGGCGGAACGTGTGGGTTCCCTTGGCCTCTGC

CCCCTAGAGGGACCCCAGTGGGGTGGGGACCAGGGGAGCCCCCACCTTGCAGCAGGCACA
||||||||||||||||||||||||||||||||||||||||||||||||||||||||||||
CCCCTAGAGGGACCCCAGTGGGGTGGGGACCAGGGGAGCCCCCACCTTGCAGCAGGCACA

GTACTTCCAGCAGAGCAGCAGCAGCAGGACCGGGAACAGGAGGAGGAAGATGAGCAGGGG
||||||||||||||||||||||||||||||||||||||||||||||||||||||||||||
GTACTTCCAGCAGAGCAGCAGCAGCAGGACCGGGAACAGGAGGAGGAAGATGAGCAGGGG

GATGAGCCACCAGAAGGTGCCCGGGGGGCATTCTAGGGAGGAGAGAGGAGGAGGAGGGAG
||||||||||||||||||||||||||||||||||||||||||||||||||||||||||||
GATGAGCCACCAGAAGGTGCCCGGGGGGCATTCTAGGGAGGAGAGAGGAGGAGGAGGGAG

CCAGTGCCTCCCCCCGATGTGGGTCTGACAGGCCACCCAGGCCCACCCTGCCAGCCCGCC
||||||||||||||||||||||||||||||||||||||||||||||||||||||||||||
CCAGTGCCTCCCCCCGATGTGGGTCTGACAGGCCACCCAGGCCCACCCTGCCAGCCCGCC

GGCTCACCCTTCCTCCTCTGCACCAAGACGGTGCTGTTGGGCCCGGGGGCGCTGTCCCCC
||||||||||||||||||||||||||||||||||||||||||||||||||||||||||||
GGCTCACCCTTCCTCCTCTGCACCAAGACGGTGCTGTTGGGCCCGGGGGCGCTGTCCCCC

TCCACGGTGTAACTGTAGGTACAGTCGTCGTCCTCGTCCCGGAAGGAGCAGTGCTCCACC
||||||||||||||||||||||||||||||||||||||||||||||||||||||||||||
TCCACGGTGTAACTGTAGGTACAGTCGTCGTCCTCGTCCCGGAAGGAGCAGTGCTCCACC

ACCTCCTCCGCTGCAGACACAGATGGTCAGCAGGGCACCCGACCCCAGCCCGGAGCACCT
||||||||||||||||||||||||||||||||||||||||||||||||||||||||||||
ACCTCCTCCGCTGCAGACACAGATGGTCAGCAGGGCACCCGACCCCAGCCCGGAGCACCT

CAGGGCCTGGCGAGGGGTCCGCTCCTCACCACAGCCTTGCTCTGGGGCCCACCCTGCCCA
||||||||||||||||||||||||||||||||||||||||||||||||||||||||||||
CAGGGCCTGGCGAGGGGTCCGCTCCTCACCACAGCCTTGCTCTGGGGCCCACCCTGCCCA

TCACAGGGTCAGGGTGGAGGGGTGGAGGCCTCTCCCTGGGGAAGAATGTGAGTTCCTGGC
|||||||||| |||||||||||||||||||||||||||||||||||||||||||||||||
TCACAGGGTCGGGGTGGAGGGGTGGAGGCCTCTCCCTGGGGAAGAATGTGAGTTCCTGGC

AGACTGTGCACACGGTGGGCGGTGAAGTGGGGGCTGCCCTGCCCAGGACCGTCAAAGCCC
||||||||||||||||||||||||||||||||||||||||||||||||||||||||||||
AGACTGTGCACACGGTGGGCGGTGAAGTGGGGGCTGCCCTGCCCAGGACCGTCAAAGCCC

GCAAATCTGTGCCTGTCCTGGGCATCACTCAGGAGCTGGGCTTCTAGAACATTTTTCTGA
||||||||||||||||||||||||||||||||||||||||||||||||||||||||||||
GCAAATCTGTGCCTGTCCTGGGCATCACTCAGGAGCTGGGCTTCTAGAACATTTTTCTGA

CCCAGAGGAAAGGAGGCTGTGTGCCCCAGGGCCCGTTTTTGCGCCTTTGCTATGTTCACG
||||||||||||||||||||||||||||||||||||||||||||||||||||||||||||
CCCAGAGGAAAGGAGGCTGTGTGCCCCAGGGCCCGTTTTTGCGCCTTTGCTATGTTCACG

GCCCCCTCCCCAGGACTGGGTGCCCTGGCCAGACTGTGCGTCCCCTCCCTGGGTCTGTGT
||||||||||||||||||||||||||||||||||||||||||||||||||||||||||||
GCCCCCTCCCCAGGACTGGGTGCCCTGGCCAGACTGTGCGTCCCCTCCCTGGGTCTGTGT
```

```
GCCCAGTCTGGATTCTGTGACCACCCCCCAGGCCTGGAACCCCTCTTCTGCCTGTCCTCA
||||||||||||||||||||||||||||||||||||||||||||||||||||||||||||
GCCCAGTCTGGATTCTGTGACCACCCCCCAGGCCTGGAACCCCTCTTCTGCCTGTCCTCA

CTTTGGACTACGAGCCCCCTACCCAGCTCTACAGCCCCGCCCTGATGAGCGACCCCCCTT
||||||||||||||||||||||||||||||||||||||||||||||||||||||||||||
CTTTGGACTACGAGCCCCCTACCCAGCTCTACAGCCCCGCCCTGATGAGCGACCCCCCTT

AGAGGTCTCCCACCCAGCTCTACAGCCCTGCCCTGATGAGTGACCCCCCTTAGAGGTCTC
|||||||||||||||||||||||||||||||||||||||| |||||||||||||||||||
AGAGGTCTCCCACCCAGCTCTACAGCCCTGCCCTGATGAGCGACCCCCCTTAGAGGTCTC

CCACCCAGCTCTACAGCCCCCCTGATGAGCGACCCCCCTTAGAGGTCTCCCACCCAGCTC
||||||||||||||||||||||||||||||||||||||||||||||||||||||||||||
CCACCCAGCTCTACAGCCCCCCTGATGAGCGACCCCCCTTAGAGGTCTCCCACCCAGCTC

TACAGCCCCCCTGATGAGCGACCCCCCTTAGA-GGTCTCCCACCCAGCTCTACAG-CCCC
|||||||||||||||||||||||||||||||| |||||||||||||||||||||| ||||
TACAGCCCCCCTGATGAGCGACCCCCCTTAGATGGTCTCCCACCCAGCTCTACAGCCCCC

CCTGATGAGCGACCCCGCTTAGGGGTCTCCCACCCAGCTCTACAGCCCCGCCCTGATGAG
||||||||||||||||||||||||||||||||||||||||||||||||||||||||||||
CCTGATGAGCGACCCCGCTTAGGGGTCTCCCACCCAGCTCTACAGCCCCGCCCTGATGAG

CGACCCCGCTTAGGGGTCTCCCACCCAGCTCTACAGCCC--CCCTGATGAGCGACCCCCC
|||||||||||||||||||||||||||||||||||||||  |||||||||||||||||||
CGACCCCGCTTAGGGGTCTCCCACCCAGCTCTACAGCCCCGCCCTGATGAGCGACCCCCC

TTAGAGGTCTCCCACCCAGCTCTACAGCCCCGCCCTGATGAGCGACCCCCCTTAGAGGTC
||||||||||||||||||||||||||||||||||||||||||||||||||||||||||||
TTAGAGGTCTCCCACCCAGCTCTACAGCCCCGCCCTGATGAGCGACCCCCCTTAGAGGTC

TCCCACCCAGCTCTACAGCCCCGCCCTGATGAGCGACCCCCCTTAGAGGTCTCCCACCCA
||||||||||||||||||||||||||||||||||||||||||||||||||||||||||||
TCCCACCCAGCTCTACAGCCCCGCCCTGATGAGCGACCCCCCTTAGAGGTCTCCCACCCA

GCTCTACAGCCCCCCTGATGAGCGACCCCCCTTAGAGGTCTCCCACCCAGCTCTACAGCC
||||||||||||||||||||||||||||||||||||||||||||||||||||||||||||
GCTCTACAGCCCCCCTGATGAGCGACCCCCCTTAGAGGTCTCCCACCCAGCTCTACAGCC

CCCCTGATGAGCGACCCCGCTTAGGGGTCGCCCCTTGCCCAGTTCTCTTGCCTGCCCTGT
||||||||||||||||||||||||||||||||||||||||||||||||||||||||||||
CCCCTGATGAGCGACCCCGCTTAGGGGTCGCCCCTTGCCCAGTTCTCTTGCCTGCCCTGT

ACCTTTCTTGAGCTCATCCACCATCTTGACCTTGAAGCTGCACTCCTCGCACGTGCGCCC
||||||||||||||||||||||||||||||||||||||||||||||||||||||||||||
ACCTTTCTTGAGCTCATCCACCATCTTGACCTTGAAGCTGCACTCCTCGCACGTGCGCCC

CTTCTTTTCACCGGTGCCCCAGGCCTGGCACTGCACGCAGGAGCGCAGGTCCTCACAGAG
||||||||||||||||||||||||||||||||||||||||||||||||||||||||||||
CTTCTTTTCACCGGTGCCCCAGGCCTGGCACTGCACGCAGGAGCGCAGGTCCTCACAGAG

GCCCAGGTGGATCTGGGTGGGCAAAGAGGAGGGGCAGACGGGTACTGTGCTGGGTGGCCC
||||||||||||||||||||||||||||||||||||||||||||||||||||||||||||
GCCCAGGTGGATCTGGGTGGGCAAAGAGGAGGGGCAGACGGGTACTGTGCTGGGTGGCCC

CTGCCAGGCCCACTCCCACAACCTCACTGCCTGGGCAGCTCCCATGGCGATGGGTCCCCG
||||||||||||||||||||||||||||||||||||||||||||||||||||||||||||
CTGCCAGGCCCACTCCCACAACCTCACTGCCTGGGCAGCTCCCATGGCGATGGGTCCCCG
```

```
TGCCCACACCCAGCGAGTGTGGCCTCACCGCCGAGTAGTTGATCTCGCAGACGGTGTCCG
||||||||||||||||||||||||||||||||||||||||||||||||||||||||||||
TGCCCACACCCAGCGAGTGTGGCCTCACCGCCGAGTAGTTGATCTCGCAGACGGTGTCCG

TGTAGAGCGACTGTTGGTTGCAGTGGCAGCGGCCGCACTCGCAGTGGCCACGCCCATTAC
||||||||||||||||||||||||||||||||||||||||||||||||||||||||||||
TGTAGAGCGACTGTTGGTTGCAGTGGCAGCGGCCGCACTCGCAGTGGCCACGCCCATTAC

AGAGACCCTGAGGCGAGAGGAGGCCCCCGTGAGCCAGCTGCGCCCCCATCCCTGTTTGCG
||||||||||||||||||||||||||||||||||||||||||||||||||||||||||||
AGAGACCCTGAGGCGAGAGGAGGCCCCCGTGAGCCAGCTGCGCCCCCATCCCTGTTTGCG

AGGGGGCCCTGCCCACCGTCCCCCACTGCCTGCCTCAGGCCCTGGCCCACCCCGTTACTG
||||||||||||||||||||||||||||||||||||||||||||||||||||||||||||
AGGGGGCCCTGCCCACCGTCCCCCACTGCCTGCCTCAGGCCCTGGCCCACCCCGTTACTG

TCGATGCAGGTGGCATTGCTGAGAGGACAGNNNNNNNNNNNNNNNNNNNNNNNNNNNNNN
||||||||||||||||||||||||||||||||||||||||||||||||||||||||||||
TCGATGCAGGTGGCATTGCTGAGAGGACAGNNNNNNNNNNNNNNNNNNNNNNNNNNNNNN

NNNNNNNNNNNNNNNNNNNNNNNNNNNNNNNNNNNNNNNNNNNNNNNNNNNNNNNNNNNN
||||||||||||||||||||||||||||||||||||||||||||||||||||||||||||
NNNNNNNNNNNNNNNNNNNNNNNNNNNNNNNNNNNNNNNNNNNNNNNNNNNNNNNNNNNN

NNNNNNNNNNACCCTGCCCATCACAGGGTCAGGGTGGAGGGGTGGAGGCCTCTCCCTGGC
||||||||||||||||||||||||||||||||||||||||||||||||||||||||||||
NNNNNNNNNNACCCTGCCCATCACAGGGTCAGGGTGGAGGGGTGGAGGCCTCTCCCTGGC

GATAAATGTGAGTTCCTGGCAGACTGTGCACACGGTGGGCGGTTTAAGTGGGGGCTGCCC
||||||||||||||||||||||||||||||||||||||||||||||||||||||||||||
GATAAATGTGAGTTCCTGGCAGACTGTGCACACGGTGGGCGGTTTAAGTGGGGGCTGCCC

TGCCCAGGACCGTCAAAGCCCGCAAATCTGTGCCTGTCCTGGGCATCACTCAGGAGCTGG
||||||||||||||||||||||||||||||||||||||||||||||||||||||||||||
TGCCCAGGACCGTCAAAGCCCGCAAATCTGTGCCTGTCCTGGGCATCACTCAGGAGCTGG

GCTTCTAGAACATTTTTCTGACCCAGAGGAAAGGAGGCTGTGTGCCCCAGGGCCCGTTTT
||||||||||||||||||||||||||||||||||||||||||||||||||||||||||||
GCTTCTAGAACATTTTTCTGACCCAGAGGAAAGGAGGCTGTGTGCCCCAGGGCCCGTTTT

TGCGCCTTTGCTATGTTCACGGCCCCCTCCCCAGGACTGGGTGCCCTGGCCAGACTGTGC
||||||||||||||||||||||||||||||||||||||||||||||||||||||||||||
TGCGCCTTTGCTATGTTCACGGCCCCCTCCCCAGGACTGGGTGCCCTGGCCAGACTGTGC

GTCCCCTCCCTGGG
||||||||||||||
GTCCCCTCCCTGGG
```

**Chr.19: Pos. 56493087  to 56493200**

```
TCTGTGTGCCCAGTCTGGATTCTGTGACCACCCCCCAGGCCTGGAACCCCTCTTCTGCCT
||||||||||||||||||||||||||||||||||||||||||||||||||||||||||||
TCTGTGTGCCCAGTCTGGATTCTGTGACCACCCCCCAGGCCTGGAACCCCTCTTCTGCCT

GTCCTCACTTTGGACTACGAGCCCCCTACCCAGCTCTACAGCCCCGCCCTGAT
|||||||||||||||||||||||||||||||||||||||||||||||||||||
GTCCTCACTTTGGACTACGAGCCCCCTACCCAGCTCTACAGCCCCGCCCTGAT
```
