# Supplementary material for: DNA-based diagnosis of rare diseases in veterinary medicine: a 4.4 kb deletion of ITGB4 is associated with epidermolysis bullosa in Charolais cattle
Source: BMC Vet Res. 2015 Mar 4;11:48. doi: 10.1186/s12917-015-0366-0 (PMC4351973; doi:10.1186/s12917-015-0366-0)
Supplement: Additional file 3: — Candidate genes for epidermolysis bullosa (EB). A list of 18 genes and their annotated position in the bovine genome. [file 12917_2015_366_MOESM3_ESM.pdf]

Gene symbol	Chr	UMD3.1/bosTau6 assembly	
		from	to
ITGA6	2	24,131,486	24,217,715
KRT5	5	27,541,428	27,547,279
FERMT1	13	48,617,012	48,665,879
PLEC1	14	2,072,781	2,087,975
EXPH5	15	18,458,482	18,573,993
LAMC2	16	65,705,535	65,772,953
LAMB3	16	75,567,714	75,610,921
PKP1	16	81,683,409	81,721,837
ITGA3	19	37,198,628	37,232,154
KRT14	19	42,430,195	42,434,559
JUP	19	42,602,485	42,628,701
ITGB4	19	56,472,338	56,506,801
TGM5	21	55,514,928	55,549,439
COL7A1	22	51,859,652	51,889,953
DST	23	3,171,426	3,688,403
DSP	23	47,670,503	47,714,288
LAMA3	24	33,072,797	33,325,034
COL17A1	26	24,848,929	24,896,345
